# Ethical, legal, regulatory, and policy issues concerning embryoids: a systematic review of the literature

**DOI:** 10.1186/s13287-023-03448-8

**Published:** 2023-08-21

**Authors:** Ana S. Iltis, Grace Koster, Emily Reeves, Kirstin R. W. Matthews

**Affiliations:** 1 Center for Bioethics, Health and Society and Department of Philosophy, Wake Forest University, Winston-Salem, NC 27106 USA; 2 Baker Institute for Public Policy, Rice University, Houston, TX 77005 USA

**Keywords:** Embryoids, Embryos, Ethics, Pluripotent stem cells, Social implications, Synthetic embryos

## Abstract

Recent advances in methods to culture pluripotent stem cells to model human development have resulted in entities that increasingly have recapitulated advanced stages of early embryo development. These entities, referred to by numerous terms such as embryoids, are becoming more sophisticated and could resemble human embryos ever more closely as research progresses. This paper reports a systematic review of the ethical, legal, regulatory, and policy questions and concerns found in the literature concerning human embryoid research published from 2016 to 2022. We identified 56 papers that use 53 distinct names or terms to refer to embryoids and four broad categories of ethical, legal, regulatory, or policy considerations in the literature: research justifications/benefits, ethical significance or moral status, permissible use, and regulatory and oversight challenges. Analyzing the full range of issues is a critical step toward fostering more robust ethical, legal, and social implications research in this emerging area and toward developing appropriate oversight.

## Background

Methods to culture stem cells to model early human development have been reported since 2014 [1, 2]. Recent advances have resulted in entities that model different stages of early embryo development—from the blastocyst stage at 5 days post-fertilization (dpf), to gastrulation at 17 dpf, to later stages of organogenesis [3–8]. These entities are referred to by numerous terms, including embryoids, synthetic embryos, gastruloids, and blastoids [9]. For ease, we will refer to them as embryoids, a general name for all types of cell models of early development.

As science and technology advance, researchers anticipate embryoids will become more sophisticated and resemble human embryos ever more closely [8, 9]. In 2022, researchers were able to grow a mouse embryoid in culture from a cell line to a synthetic embryo that had the early formation of organs and limbs [10]. This new technology, which has only been used on mouse cells, raises concerns regarding how far scientists can and should grow human embryos and embryoids in culture [8, 9].

Embryoid, embryo, and human–animal chimera research raise a number of sometimes-overlapping ethical, regulatory, and policy issues, though they merit separate attention. Numerous scholars have written about ethical questions or concerns associated with embryoid research or that might arise as the science of embryoids advances [11, 12]. Others have highlighted policy or regulatory issues that such research prompts both regarding the status of embryoid research and the relationship between such research and human embryo research. Two recent systematic reviews examined ethical issues associated with organoids [11, 12]. We know of no systematic review regarding ethical, legal, regulatory, and policy issues regarding embryoids. Analyzing the full range of issues identified in the literature pertaining to research on human embryoids (or any stem cell-based models of early human development, regardless of the names used to describe them) is an important step toward fostering more robust ethical, legal, and social implications research in this area and developing appropriate oversight.

## Methods

This systematic review followed PRISMA reporting guidelines [13, 14]. The protocol for this paper was published in Open Science Forum and registered on June 16, 2022. In consultation with reference librarians from the Z. Smith Reynolds Library at Wake Forest University, J. Denice Lewis and Kathy Shields, we designed a search strategy that included three databases: PubMed, Embase, and Web of Science. The search strategy for each database is in Appendix 1. No date limits were used. Language limits were applied to restrict publications to English. All searches were completed on January 10, 2022. Searches were imported into Rayyan for de-duplication and then imported to Zotero. Through consultation with authors and review of reference sections of included publications, additional possible publications were identified.

To be eligible, publications had to:

1. Be discoverable using our search strategy or identified by an author;
2. Be published in English;
3. Be accessible via full text to us either online, through the Wake Forest University or Rice University Library, or through Interlibrary Loan; and
4. Identify at least one ethical, regulatory, or policy question or issue related to research involving embryoids (or any term used to describe a stem cell-based model of human embryos).

Consistent with published recommendations for systematic reviews, we included abstracts and dissertations [15, 16]. We also included commentaries, editorials, and other types of publications to maximize data collection.

### Selection

**Fig. 1:**
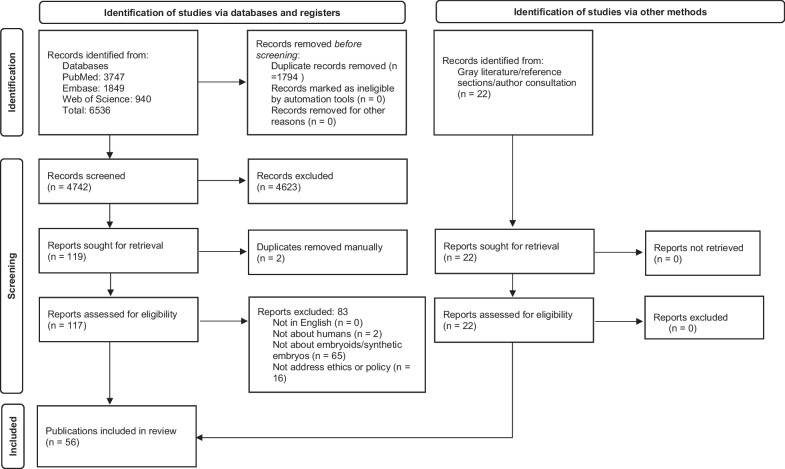
PRISMA flow diagram [92]

Following PRISMA reporting guidelines, Fig. 1 presents the selection process [13, 14]. Initial screening of titles and abstracts was done by one author (ASI) using Rayyan web-based systematic review software. If the abstract did not clearly indicate that an article should be excluded, it was tagged as a “Maybe” and advanced to the next round. In the second phase, three authors (ASI, GK, ER) screened 20 of the same publications to promote consistency and inter-rater reliability. Assessments were compared after screening 10 entries and differences discussed. The same process was repeated with 10 more entries. After that, two authors (either ASI and GK or ASI and ER) screened all remaining titles and abstracts. All articles were excluded based on the highest ranking exclusion criterion in our exclusion hierarchy (see Table 1), included, or tagged “Maybe” for full-text screening. We obtained full texts for all publications marked “Maybe” or “Include.” Full texts were stored and reviewed in Zotero. Two authors (either ASI and GK or ASI and ER) reviewed all full-text publications and either included or excluded each one. Where necessary, we consulted with other authors to make inclusion and exclusion decisions. At each stage, all differences were resolved through discussion and review of the material, allowing us to have 100% consensus. Two duplicates that had not been previously detected were found during the screening process and removed manually.

**Table 1:** Exclusion hierarchy

1	Not in English
2	Not available in full text to us online, through the Wake Forest University Library, Rice University Library, or through Interlibrary Loan
3	Did not address human embryoids or synthetic embryos
4	Did not identify at least one ethical, regulatory, or policy question or issue related to research involving embryoids (or any term used to describe a stem cell-based model of human embryos)

The reference section of each included publication was reviewed for additional publications to assess. Authors were invited to recommend additional reports or publications for screening. These were screened by two authors (either ASI and GK or ASI and ER), and often in consultation with KRWM, they were marked for inclusion or exclusion.

### Data extraction and synthesis

For each included publication, data were extracted independently by two authors (either ASI and GK or ASI and ER) and entered into a data extraction form created using Google Sheets. Data items extracted were:

- Complete citation
- Publication type
- Year published
- Terms used to refer to embryoids
- Background information
- Ethical issues identified
- Commentary on ethical issues
- Policy or Regulatory issues identified
- Commentary on policy or ethical issues

Three authors (ASI, GK, and ER) reviewed the extracted data to combine information, resulting in one comprehensive data sheet. The results were shared with all authors. Through qualitative content analysis, authors identified the themes and identified categories and sub-categories reported here [17].

## Results

The initial search yielded 6536 publications. These were imported into Rayyan for de-duplication. After removing 1794 duplicates, 4472 records were screened. After screening titles and abstracts, 4623 publications were excluded because they did not address human embryoids or synthetic embryos. These articles concerned animal models or addressed only “embryoid bodies,” “organoids,” or other models that were not models of human embryos. Full texts were retrieved for the remaining 119 publications. Two additional duplicates were found and removed manually, leaving 117 publications for full-text screening. These were added to Zotero, and full texts were obtained. Of those 117 publications, 83 were excluded because they were not about human models (*n* = 2), they were not about embryoids (*n* = 65) or they did not address any ethical, regulatory, oversight, or policy issues (*n* = 16), and 34 were included. The references of the 34 included publications were reviewed for additional publications to screen, and all authors were invited to share information regarding other possible publications or reports to consider. An additional 22 publications were identified this way and screened. All 22 met our inclusion criteria. A total of 56 publications were included in the review. One of these was an erratum for another included publication [18, 19]. Included publications are listed in Table 2.

**Table 2:** Included publications

References	Publication
[18]	Aach J, Lunshof J, Iyer E, Church GM. Addressing the ethical issues raised by synthetic human entities with embryo-like features. eLife. 2017;6:e20674
[19]	Aach J, Lunshof J, Iyer E, Church GM. Correction: Addressing the ethical issues raised by synthetic human entities with embryo-like features eLife. 2017;6:e27642
[21]	Ankeny RA, Munsie MJ, Leach J. Developing a Reflexive, Anticipatory, and Deliberative Approach to Unanticipated Discoveries: Ethical Lessons from iBlastoids. Am J Bioeth AJOB. 2022 Jan;22(1):36–45
[56]	Barnhart AJ, Dierickx K. A RAD Approach to iBlastoids with a Moral Principle of Complexity. Am J Bioeth AJOB. 2022 Jan;22(1):54–6
[38]	Bartfeld S. Realizing the potential of organoids-an interview with Hans Clevers. J Mol Med Berl Ger. 2021 Apr;99(4):443–7
[40]	Boers SN, van Delden JJM, Bredenoord AL. Organoids as hybrids: ethical implications for the exchange of human tissues. J Med Ethics. 2019 Feb;45(2):131–9
[22]	Brivanlou AH, Rivron N, Gleicher N. How will our understanding of human development evolve over the next 10 years. Nat Commun. 2021 Jul 29;12(1):4614
[53]	Chan S. How and Why to Replace the 14-Day Rule. Curr Stem Cell Rep. 2018 Sep 1;4(3):228–34
[47]	Clark AT, Brivanlou A, Fu J, Kato K, Mathews D, Niakan KK, et al. Human embryo research, stem cell-derived embryo models and in vitro gametogenesis: Considerations leading to the revised ISSCR guidelines. Stem Cell Rep. 2021 Jun;16(6):1416–24
[44]	Daly T. Synthetic Human Entities with Embryo-like Features (SHEEFS) and the Incarnation. Ethics Medics. 2019;35(2):93–105
[49]	Denker HW. Autonomy in the Development of Stem Cell-Derived Embryoids: Sprouting Blastocyst-Like Cysts, and Ethical Implications. Cells. 2021 Jun 10;10(6)
[54]	Denker HW. Self-Organization of Stem Cell Colonies and of Early Mammalian Embryos: Recent Experiments Shed New Light on the Role of Autonomy vs. External Instructions in Basic Body Plan Development. Cells. 2016 Oct 25;5(4)
[63]	Denker HW. Human embryonic stem cells: the real challenge for research as well as for bioethics is still ahead of us. Cells Tissues Organs. 2008;187(4):250–6
[50]	Denker HW. Stem Cell Terminology and ‘Synthetic’ Embryos: A New Debate on Totipotency, Omnipotency, and Pluripotency and How It Relates to Recent Experimental Data. Cells Tissues Organs. 2014;199(4):221–7
[23]	Haase K, Freedman BS. Once upon a dish: engineering multicellular systems. Dev Camb Engl. 2020 May 4;147(9)
[24]	Haniffa M, Taylor D, Linnarsson S, Aronow BJ, Bader GD, Barker RA, et al. A roadmap for the Human Developmental Cell Atlas. Nature. 2021 Sep;597(7875):196–205
[67]	Hengstschläger M, Rosner M. Embryoid research calls for reassessment of legal regulations. Stem Cell Res Ther. 2021 Jun 19;12(1):356
[57]	Hurlbut JB, Hyun I, Levine AD, Lovell-Badge R, Lunshof JE, Matthews KRW, et al. Revisiting the Warnock rule. Nat Biotechnol. 2017 Nov;35(11):1029–42
[58]	Hyun I. Engineering Ethics and Self-Organizing Models of Human Development: Opportunities and Challenges. Cell Stem Cell. 2017 Dec 7;21(6):718–20
[60]	Hyun I, Munsie M, Pera MF, Rivron NC, Rossant J. Toward Guidelines for Research on Human Embryo Models Formed from Stem Cells. Stem Cell Rep. 2020;14(2):169–74
[70]	Kaebnick GE. Toward Public Bioethics? Hastings Cent Rep. 2017 May;47(3):2
[25]	Kagawa H, Javali A, Khoei HH, Sommer TM, Sestini G, Novatchkova M, et al. Human blastoids model blastocyst development and implantation. Nature. 2021 Dec 2;601:600–605
[66]	Lopes M, Truog R. The Emergence of Embryo Models in Research: Ethical Considerations. Harvard Health Policy Review. 2018; April 27
[69]	Lovell-Badge R, Anthony E, Barker RA, Bubela T, Brivanlou AH, Carpenter M, et al. ISSCR Guidelines for Stem Cell Research and Clinical Translation: The 2021 update. Stem Cell Rep. 2021 Jun 8;16(6):1398–408
[39]	Lysaght T. Anticipatory Governance and Foresight in Regulating for Uncertainty. Am J Bioeth. 2022 Jan 2;22(1):51–3
[26]	Mantziou V, Baillie-Benson P, Jaklin M, Kustermann S, Arias AM, Moris N. In vitro teratogenicity testing using a 3D, embryo-like gastruloid system. Reprod Toxicol Elmsford N. 2021 Oct;105:72–90
[55]	Matthews KRW, Iltis AS, de Melo-Martin I, Robert JS, Wagner DS. Moving the Line? Findings and Recommendations for Human Embryo Research.:13
[27]	Matthews KRW, Iltis AS, Marquez NG, Wagner DS, Robert JS, Melo-Martín I de, et al. Rethinking Human Embryo Research Policies. Hastings Cent Rep. 2021 Jan 1;51(1):47–51
[28]	Matthews KR, Moralí D. National human embryo and embryoid research policies: a survey of 22 top research-intensive countries. Regen Med. 2020 Jul;15(7):1905–17
[68]	Matthews KRW, Robert JS, Iltis AS, de Melo-Martin I, Wagner DS. Cell-Culture Models of Early Human Development: Science, Ethics, and Policy. 2019. Rice University Baker Institute for Public Policy
[9]	Matthews KRW, Wagner DS, Warmflash A. Stem cell-based models of embryos: The need for improved naming conventions. Stem Cell Rep. 2021;16(5):1014–20
[48]	Monasterio Astobiza A, Molina Pérez A. Why iBlastoids (Embryo-like Structures) Do Not Rise Significant Ethical Issues. Am J Bioeth AJOB. 2022 Jan;22(1):59–61
[59]	Mummery C, Anthony E. New guidelines for embryo and stem cell research. Nat Rev Mol Cell Biol. 2021 Dec;22(12):773–4
[43]	Munsie M, Gyngell C. Ethical issues in genetic modification and why application matters. Curr Opin Genet Dev. 2018 Oct;52:7–12
[51]	Munsie M, Hyun I, Sugarman J. Ethical issues in human organoid and gastruloid research. Dev Camb Engl. 2017 Mar 15;144(6):942–5
[41]	Nicolas P, Etoc F, Brivanlou AH. The ethics of human-embryoids model: a call for consistency. J Mol Med. 2021 Apr 1;99(4):569–79
[46]	Pera M. Embryogenesis in a dish. Science. 2017;356(6334):137–8
[45]	Pera MF, de Wert G, Dondorp W, Lovell-Badge R, Mummery CL, Munsie M, et al. What if stem cells turn into embryos in a dish? Nat Methods. 2015;12(10):917–9
[62]	Pereira Daoud AM, Popovic M, Dondorp WJ, Trani Bustos M, Bredenoord AL, Chuva de Sousa Lopes SM, et al. Modelling human embryogenesis: embryo-like structures spark ethical and policy debate. Hum Reprod Update. 2020 Nov 1;26(6):779–98
[64]	Piotrowska M. Research guidelines for embryoids. J Med Ethics. 2021;47:e67
[61]	Piotrowska M. Avoiding the potentiality trap: thinking about the moral status of synthetic embryos. Monash Bioeth Rev. 202,038(2):166–80
[65]	Pullicino P, Richard EJ, Burke WJ. Mass Production of Human “Embryoid” Cells from Developmentally Frozen Embryos: Is It Ethical? Linacre Q. 2020;87(3):347–50
[52]	Rao H. How to Conduct Ethical Research on SHEEFs: Biological Background, the Classification, and Recommendations for Guideline Development on These New Synthetic Embryos. 2019. ProQuest. Wake Forest University
[37]	Regalado A. Meet the “artificial embryos” being called uncanny and spectacular. MIT Tech Rev. 2019
[29]	Rosner M, Reithofer M, Fink D, Hengstschläger M. Human Embryo Models and Drug Discovery. Int J Mol Sci. 2021;22(2)
[30]	Rossant J. Gene editing in human development: ethical concerns and practical applications. Dev Camb Engl. 2018;145(16)
[31]	Shao Y, Fu J. Synthetic human embryology: towards a quantitative future. Curr Opin Genet Dev. 2020;63:30–5
[71]	Kaebnick GE. Toward Public Bioethics? Hastings Cent Rep. 2017;47(3):2
[72]	Subbaraman N. Research on embryo-like structures struggles to win US government funding. Nature. 2020;577(7791):459–60
[32]	Tomoda K, Hu H, Sahara Y, Sanyal H, Takasato M, Kime C. Reprogramming epiblast stem cells into pre-implantation blastocyst cell-like cells. Stem Cell Rep. 2021;16(5):1197–209
[42]	Tomoda K, Kime C. Synthetic embryology: Early mammalian embryo modeling systems from cell cultures. Dev Growth Differ. 2021;63(2):116–26
[33]	van den Brink SC, van Oudenaarden A. 3D gastruloids: a novel frontier in stem cell-based in vitro modeling of mammalian gastrulation. Trends Cell Biol. 2021;31(9):747–59
[34]	Weatherbee BAT, Cui T, Zernicka-Goetz M. Modeling human embryo development with embryonic and extra-embryonic stem cells. Dev Biol. 2021;474:91–9
[73]	Wei Y, Zhang C, Fan G, Meng L. Organoids as Novel Models for Embryo Implantation Study. Reprod Sci. 2021;28(6):1637–43
[35]	Wilger K. Gaps in Embryo Model Ethics. Ethics Medics. 2020;45(10):1–4
[36]	Williams K, Johnson MH. Adapting the 14-day rule for embryo research to encompass evolving technologies. Reprod Biomed Soc Online. 2020;10:1–9

We identified 53 distinct names or terms used to refer to embryoids (see Table 3). There are three different types of terms used to identify embryoids previously described: general, time-based, and cell-based [9]. Some terms, such as embryoid or cell-based embryo model, describe the field as a whole. Other terms, such as blastoid or gastruloid, identify a subset of entities at a specific biological moment that they are recapitulating, the blastocyst or gastrulation, respectively. Still other terms identify the cell types used in the model, for example, ETX embryos which describe embryoids using embryonic, trophoblast, and extra-embryonic endoderm stem cells [20].

**Table 3:** Names identified

Term	References
2C-like cells	[54]
3D blastocyst culture system	[73
3D embryo-like gastruloid system	[26]
3D structures that resemble pre-implantation embryos	[39]
Artificial embryos	[28, 37, 66]
Asymmetric human epiblast	[29, 49]
Blastoids	[9, 25, 28, 33, 35, 42, 49, 59, 68, 73]
Embryo models	[22, 35, 36, 64, 72]
Embryo-like entities	[37, 44, 66, 69]
Embryo-like structures	[29, 48, 61, 72]
Embryoid bodies	[68]
Embryoids	[9, 27–30, 32, 36–38, 41, 42, 49, 55, 64–68, 70]
ETC embryoids	[41]
ETS/ETX embryos	[9, 33]
Gastrulation micropatterned colony	[29]
Gastruloids	[9, 23, 26, 28–30, 33–35, 41, 43, 45, 46, 49, 51, 53, 58, 59, 61, 68]
Gastrulating embryo-like structures	[49]
Human cell culture models	[52]
Human cell cultures of early development (hCCMEDs)	[55, 68]
Human embryo-like structures derived from pluripotent stem cells	[59, 62]
Human embryoid model	[41]
Human epiblast models	[33]
Iblastoid	[21, 39, 48, 56]
Integrated models of human development	[47, 49, 59]
Micropatterned 2D culture systems (2D gastruloids)	[33]
Micropatterned cultures of human pluripotent stem cells	[30]
Micropatterned hESC colonies	[9, 28, 68]
Micropatterned stem cell cultures	[41]
Models of early human development	[59]
Model of the human blastocyst	[25]
Multiple human embryo-like cells (MPECs)	[65]
Non-integrated models of human development	[47, 49, 59]
Organized embryo-like structures	[47]
Organoids/embryonic organoids	[9, 51, 70, 73]
PSC-derived models of early embryo development	[28]
Polarized embryo-like structures	[49]
Post-implantation amniotic sac embryoids (PASE)	[9, 28, 29, 33, 49]
Post-implantation epiblast	[29]
Self-organizing hESCs	[52]
Self-organizing models of human development	[58]
Self-organizing stem cell model systems (SOSCS)	[48]
Synthetic embryo-like structures	[40, 71]
Synthetic human entities with embryo-like features (SHEEFS)	[9, 18, 28, 36, 44, 49, 52, 53, 61, 68, 70]
Stem cell-based models of embryos	[9]
Stem cell-derived blastoids	[47]
Stem cell-derived models of embryo development	[69]
Stem cell-based embryo models/stem cell-based models of embryos	[9, 24, 34, 47, 49, 69]
Structures that resemble embryos	[45, 46]
Synthetic embryo-like entities	[57]
Synthetic embryo systems (SES)	[32, 42]
Synthetic embryos	[9, 23, 29, 35, 37, 44, 50, 52, 55, 61, 62, 66, 68, 71]
Synthetic embryoids	[71]
Synthetic entities with embryo-like features	[29, 37]

**Fig. 2:**
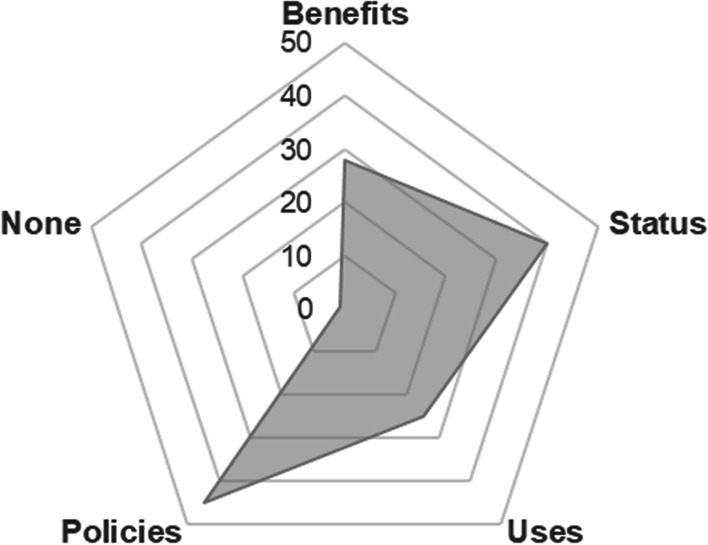
Major themes identified

Through an iterative inductive process, we identified four broad categories of ethical, legal, regulatory, or policy considerations found in the literature, each of which is discussed in more detail below: research justifications/potential benefits, ethical significance or moral status, permissible use, and regulatory and oversight challenges. As depicted in Fig. 2, the majority of papers included discussions about oversight, policies and regulations (Policies, *n* = 45), and the ethical significance or moral status of embryoids (Status, *n* = 40). Fewer publications discussed potential benefits (Benefits, *n* = 28) and uses and applications of embryoid research (Uses, *n* = 25). One publication stated that embryoid research raises no ethical or regulatory considerations (None, *n* = 1).

### Justifications and potential benefits of embryoid research

Some authors noted that embryoid research requires justification and many indicated that potential benefits associated with the research could justify it (*n* = 28) [9, 21–47]. Over one-third of the publications (*n* = 19) noted that embryoid research could avoid some of the ethical concerns or practical problems associated with human embryo research [9, 21–37, 47]. Several scholars also believed that embryoid research could offer an ethical alternative to animal research and reduce reliance on animals (*n* = 8) [22, 26, 30, 31, 35, 38, 39, 47]. A third potential benefit noted is that embryoid research could improve scientific knowledge in ways that advance human health (*n* = 12) [9, 21, 22, 24, 26, 30, 36, 40–42, 45, 46]. Specific examples of potential health benefits include knowledge that could improve understanding of early pregnancy loss, management of early pregnancy, and treating early developmental disorders [22]. Although embryoid research can raise concerns over the destruction of human embryos, it was suggested that when scientists use induced pluripotent stem cells (which are derived from adult cells) rather than embryonic stem cells, they can avoid these issues (*n* = 3) [9, 29, 33]. Interestingly, the view that embryoid research requires justification based on potential benefits might suggest that such research raises ethical considerations. This contrasts with the view expressed in one publication that embryoid research raises no ethical considerations: “iBlastoids [embryo-like structures] do not pose any serious ethical concern for several reasons and would not need a robust ethical framework that thoughtfully foresees unintended and unanticipated consequences” [48]. The full list of potential benefits that could justify embryoid research are also summarized in Table 4.

**Table 4:** Main ethical, legal, regulatory, and policy issues and themes identified in journal articles

Issues	References
*Potential benefits*	
Avoids practical and ethical concerns human embryo research raises	[9, 21–37, 47]
Reduces the reliance on animals for research	[22, 26, 30, 31, 35, 38, 39, 47]
Yields important knowledge to improve human health	[9, 21, 22, 24, 26, 30, 36, 40–42, 45, 46]
Eliminates the destruction of human embryos when induced pluripotent stem cells are used	[9, 29, 33]
*Ethical significance or moral status concerns*	
Potential to create synthetic human life	[38, 49–52]
Are embryoids clones and, if so, what follows?	[21, 39, 45, 48, 52, 53, 61, 62, 65, 69]
Varying assessment of embryo moral status result in varying assessments of embryoid moral status	[18, 21, 28, 38, 41, 55, 61, 62, 77]
Assessment of moral status of embryos based on morally relevant features could guide assessment of embryoid moral status	[18, 30, 31, 37, 41, 44, 57, 63, 64]
Ethical significance of embryoids may vary based on their features	[9, 47, 49, 55]
Need to determine which features are morally relevant for assessing moral status	[49, 53, 54]
Level of embryoid complexity could determine moral status	[41, 49, 51, 54, 56–60]
Possibility of embryoids experiencing pain could determine moral status	[18, 36, 43, 44, 53, 57, 61]
Potential sentience in embryoids could determine moral status	[18, 36, 43, 57, 62]
Human organismic potential of embryoids determines moral status	[9, 18, 21, 23, 26, 35–37, 41, 49, 50, 52–54, 59–66]
What is the proper relationship between treatment of embryos and embryoids?	[9, 37, 41, 55, 57, 60, 61]
Embryoid research involves embryo destruction	[28, 68]
*Permissible uses or applications*	
Embryoid research requires some limits	[34, 50, 51]
Should reproductive use should be banned?	[22, 37, 39, 47, 48, 52, 53, 60, 67, 69]
Should chimera creation should be banned?	[18, 52, 53]
Limit-setting may not be possible	[49]
Resolving questions regarding embryoids’ moral status may not resolve questions regarding permissible use	[41, 55, 62]
Are researcher intentions relevant to assessing permissibility of research?	[21, 35, 37, 41, 48, 57, 61]
Embryoid research could involve commercialization of human tissue	[40]
*Regulatory and policy considerations*	
Questions regarding how embryoids fit within existing research guidelines, policies, and frameworks	[9, 18, 21, 23, 27, 28, 33, 34, 41, 44, 50–52, 55, 57, 60, 62, 64, 66, 67, 70–72]
Embryoids do not follow canonical embryogenesis	[9, 18, 27, 31, 34, 37, 47, 53, 60, 66, 67, 69, 70]
Implications for the 14-day limit for human embryo research	[18, 27, 41, 49, 54, 62, 64]
Inconsistent definitions of embryos and fetuses in existing regulations, policies, laws, and guidelines	[41, 45, 46, 55, 57, 60, 62, 64, 67]
Effect on stem cell research policy or oversight	[56, 67]
Should embryoid research be subject to separate regulations, guidelines, and oversight practices?	[18, 21, 26–29, 34, 44, 47, 52, 55, 67]
New policies and application of existing frameworks to embryoid research could undermine embryoid research	[39, 41, 70]
What should be the goals and priorities of embryoid research oversight and regulation?	[9, 18, 21, 41, 51, 59–61, 66]
Public trust in science or scientific institutions must be maintained	[43, 52, 55]
Transparency of science should be promoted	[21, 27, 41, 69]
Should the moral status of the embryoids determine policy adopted?	[53]
Should embryoid policy follow the precautionary principle?	[39]
Should different types of embryoids be subject to different regulations?	[41]
Who should develop any new guidelines, regulations, or policies and how?	[21, 27, 42, 52, 55, 57, 59, 60, 66]
Are public consultation, engagement, or deliberation necessary for developing guidelines?	[18, 21, 27, 36, 39, 42, 45, 46, 53, 60, 70]
International collaboration is important for developing guidelines	[53]
Ethical framework for embryoid research should be developed before regulatory framework	[18, 22, 39, 47, 54, 67]
Informed consent should be addressed in new guidelines	[43, 51, 64, 73]
Privacy should be addressed in new guidelines	[61, 73]
Benefit sharing should be addressed in new guidelines	[73]
New regulations, guidelines, or policies should be flexible to adapt to evolving science	[21, 23, 39, 41, 56, 59, 60, 67]

### Ethical significance or moral status of embryoids

Several authors mentioned issues related to assessing the ethical significance or moral status of embryoids (summarized in Table 4). At the most general level, some authors (*n* = 5) noted that the possibility of creating synthetic human life raises concerns and suggested that embryoids are or could become sufficiently complex that they constitute synthetic human life [38, 49–52]. Three publications noted the importance of determining the features of an entity that are morally relevant and how those features relate to an entity’s moral status [49, 53, 54]. A related claim was that there could be ethically significant differences among different types of embryoids based on their features (*n* = 4) [9, 47, 49, 55]. Many other publications addressed the issue of morally relevant features in more detail by addressing specific features. Some authors noted that the level of complexity of embryoids would affect the ethical issues such research raises (*n* = 9) [41, 49, 51, 54, 56–60]. Specific morally relevant or potentially morally relevant features identified in the publications reviewed included the possibility of experiencing pain (*n* = 7) [18, 36, 43, 44, 53, 57, 61], the possibility of sentience (*n* = 5) [18, 36, 43, 57, 62], and human organismic potential (*n* = 22) [9, 18, 21, 23, 26, 35–37, 41, 49, 50, 52–54, 59–66].

In addition, authors discussed the ethical significance or status of embryoids in relation to human embryos and clones. According to some authors, the ethical significance or status of embryoids could be assessed by first determining which features of embryos are morally relevant and then determining which of those features also appear in embryoids (*n* = 9) [18, 30, 31, 37, 41, 44, 57, 63, 64]. Many publications addressed the relationship between ethical assessments of embryos and embryoids. Some suggested that different accounts of the moral significance of human embryos likely would result in different assessments of embryoids (*n* = 9) [18, 21, 28, 38, 41, 55, 61, 62, 67]. Several publications raised the question of whether embryos and embryoids should be treated the same or differently (*n* = 7) [9, 37, 41, 55, 57, 60, 61]. Two publications noted that embryoid research might lead to reconsideration of the question of what respect is owed to embryos [54, 63]. Another issue that draws on the connection between embryo and embryoid research was the observation that embryoid research could raise the same concerns that human embryonic stem cell (hESC) research raises insofar as both involve embryo destruction (*n* = 2) [28, 68]. A final set of issues regarding the ethical significance of embryoids was the question of whether they are clones and, if so, what concerns that might raise (*n* = 10) [21, 39, 45, 48, 52, 53, 61, 62, 65, 69].

### Permissible uses of embryoid research

A third category of ethical issues concerns permissible uses or applications of embryoid research (summarized in Table 4). While several publications noted the need to set some limits on the use of embryoids (*n* = 3) [34, 50, 51], one publication questioned whether it would be possible to effectively draw lines limiting such research [49]. Three publications noted that while much attention is paid to the uncertain moral status of embryoids, resolving those questions will not necessarily resolve the question of whether embryoid research is permissible and, if so, which research is permissible (*n* = 3) [41, 55, 62]. Other articles questioned whether the intentions of researchers, such as the absence of reproductive intentions, were relevant to assessing the permissibility of embryoid research (*n* = 7) [21, 35, 37, 41, 48, 57, 61]. One publication raised concerns regarding the commercialization of human tissue [40]. Finally, some authors raised questions about banning the use embryoids for reproduction (*n* = 10) [22, 37, 39, 47, 48, 52, 53, 60, 67, 69] or to create chimeras (*n* = 3) [18, 52, 53].

### Regulatory, and policy considerations regarding embryoid research

A significant number of publications addressed issues related to regulations, oversight mechanisms, guidelines, or policies pertaining to embryoid research (Table 4). These fell into three sub-categories. The first sub-category concerns the relationship between regulation or oversight of embryoid research and previously existing guidelines for human embryo and hESC research. A large group of publications acknowledge that existing human embryo research and cloning laws, policies, and regulations have unclear implications for embryoid research (*n* = 23) [9, 18, 21, 23, 27, 28, 33, 34, 41, 44, 50–52, 55, 57, 60, 62, 64, 66, 67, 70–72]. Some stated that the regulations do not apply directly, creating a regulatory gap (*n* = 3) [41, 60, 62]. Scholars suggest that the major reason for a regulatory gap is that embryoids do not follow canonical embryogenesis, making references to the 14-day rule or appearance of the primitive streak irrelevant (*n* = 13) [9, 18, 27, 31, 34, 37, 47, 53, 60, 66, 67, 69, 70].

Others were concerned with how embryoid research impacts for regulations, guidelines, policies, or oversight practices of human embryo and hESC research. Several publications indicated that embryoid research motivates revisiting the 14-day rule (*n* = 6) [18, 41, 49, 54, 62, 64] or has implications for the 14-day rule (*n* = 4) [18, 27, 41, 54] that has governed human embryo research for more than 40 years. Two publications noted that future decisions regarding embryoid research oversight or regulation could have implications for other types of stem cell research [65, 67]. Finally, several publications noted the many different definitions of embryos and fetuses used in existing policies, guidelines, laws, and regulations, and authors suggested that embryoid research points to the need to revisit those definitions (*n* = 9) [41, 45, 46, 55, 57, 60, 62, 64, 67].

The second sub-category concerns development of new guidelines, policies, regulations, or oversight mechanisms for embryoid research. A group of publications raised questions regarding the need for separate ethical guidelines, oversight procedures, or regulatory framework for embryoid research (*n* = 12) [18, 21, 26–29, 34, 44, 47, 52, 55, 67]. Insofar as separate ethical and regulatory frameworks are necessary, several publications addressed the overall goals and priorities that should inform them (*n* = 9) [9, 18, 21, 41, 51, 59–61, 66]. Some noted that an ethical framework for embryoid research should first be developed and then appropriate regulations and oversight procedures should be based on that framework (*n* = 6) [18, 22, 39, 47, 54, 67]. One publication raised the question of whether and how judgments about the moral status of embryoids should shape a regulatory framework and of how policies, guidelines, and regulations should treat entities whose ontological status is unclear [53]. One raised the question of how differences among embryoid types should inform regulation [41]. Another questioned the appropriate role of the precautionary principle in shaping policy and practice [39]. Several possible overarching goals or concerns regarding the development of guidelines, policies, regulations, or oversight practices were noted. One concern was that implementing new policies or applying existing rules and regulations to embryoid research could undermine important research (*n* = 3) [39, 41, 70]. Several publications noted the importance of maintaining public trust in science and scientific institutions (*n* = 3) [43, 52, 55], or promoting transparency about science (*n* = 4) [21, 27, 41, 69].

The third sub-category concerns the scope of any new policies, guidelines, regulations, or oversight mechanisms that might developed. Various publications addressed questions of how they should be developed and who should be involved (*n* = 9) [21, 27, 42, 52, 55, 57, 59, 60, 66]. In particular, one publication noted the importance of including different perspectives and securing international collaboration to avoid disrupting science [53]. Many publications indicated that public consultation, engagement, and deliberation were necessary for the process to be legitimate (*n* = 11) [18, 21, 27, 36, 39, 42, 45, 46, 53, 60, 70].

There were also several publications that addressed the scope of new guidelines, policies, oversight mechanisms, or regulations. According to some authors, they should address donor rights and interests such as informed consent for gamete or embryo donors (*n* = 4) [43, 51, 64, 73]; privacy (*n* = 3) [51, 61, 73]; and benefit sharing (*n* = 1) [73]. Publications also noted that given the evolving nature of embryoid research, any regulations, guidelines, or policies would need to be sufficiently flexible to adapt to changes in science (*n* = 8) [21, 23, 39, 41, 56, 59, 60, 67].

## Discussion

Systematic review of ethical concerns, questions, reasons, and arguments regarding emerging technologies and practices often reveals additional questions for investigation and can help to advance ethical research, guideline development, and practice by providing an overview of the relevant issues [11, 12, 74, 75]. Our findings reveal several areas for further assessment regarding embryoid research.

### Names and definitions

There is no consensus regarding the term that should be used to identify these new entities [9]. Scientists have used both broad and specific names in their publications. Some use complicated jargon-laden names that refer to what these entities are scientifically and what they are derived from. For example, a 2019 paper created a system to make entities that “recapitulate developmental events reflecting epiblast and amniotic ectoderm development in the post-implantation human embryo” [6]. This approach raises at least two concerns. First, it likely makes it more challenging for non-experts to understand what was created, rendering science less rather than more transparent. Second, similar general names are used often for both two- and three-dimensional embryoids, which have significantly different potential to recapitulate an embryo faithfully and precisely.

Decisions about what to call embryoids are important for multiple reasons. First, names and descriptions can affect ethical perceptions of embryoids, a point that others have made regarding organoids [76]. For instance, the term “synthetic human embryo” could immediately generate concerns about destruction of these entities or the possibility of gestating these entities to live birth. In contrast, a term such as gastruloid is much less likely to generate those questions, particularly among the lay public that would not associate it with gastrulation or an early embryo. Second, names can have implications for funding, oversight, and ethical assessment. For instance, if they are referred to in terms of their stem cell origin, oversight of such research might be delegated to stem cell research oversight committees. Using the term synthetic human embryos might trigger review by a committee overseeing embryo research. Depending on how they are described and treated, embryoid research could face different funding or other restrictions. For instance, ISSCR categorizes embryoids into two types: “integrated” embryoids that include all cell or tissue types (and which are to receive full reviews) and “non-integrated” embryoids, which are missing extra-embryonic cells or tissues (which do not require full reviews and instead only needed to be reported to an oversight committee) [47]. Researchers can ensure that their work is viewed as non-integrated and receives reduced oversight by specifying the lack of a cell-type.

There is also no consistent definition of embryoids [9]. No criteria that an entity must meet to be considered an embryoid have been established. There are no shared mechanisms for differentiating between simpler models and more sophisticated models that have greater capacity to develop more fully. Other than the distinction between “integrated” and “non-integrated” embryoids that some scientists use, no additional work to understand what embryoids are has been conducted [9, 47].

It may be helpful to think of questions regarding which entities should be classified as embryoids in terms of long-standing debates in the philosophy of science regarding essentialist, pluralist, and cluster concept approaches to classifying species. One possibility is that we must identify a list of necessary and sufficient conditions an entity must meet to be an embryoid. Attempting to articulate the essence of what is an embryoid could prove impossible, much as essentialist approaches to defining species have faced serious challenges and fallen out of favor [77, 78]. Alternatively, there might be multiple different ways to think about when an entity is an embryoid. Although this still would require identifying the plurality of ways such entities might be classified, it would alleviate the need to identify a single set of criteria, which is what the many varieties of pluralist accounts of species definition offer biologists [77–79]. Finally, borrowing from Wittgenstein, “embryoid” might be a cluster concept [80]. The entities share a family resemblance because of various properties that they have without having to possess all of the properties to “count,” much as some philosophers of science have suggested is the case for the concept “species” [81]. Defining the “complicated network of similarities overlapping and criss-crossing” (Wittgenstein §66) that are pertinent to embryoids requires further analysis and re-assessment as research advances.

In establishing criteria for embryoids, important questions related to embryos likely will surface. These include questions about when an embryoid is sufficiently similar to an embryo to be treated as such and, in turn, how embryos ought to be treated, issues we address below.

### Fundamental philosophical questions

Numerous metaphysical, epistemological, and ethical questions associated with human embryo research continue to be debated [27]. Embryoids raise many questions similar to those associated with embryo research as well as new ones. We expect disagreements and uncertainties comparable to the human embryo and hESC research debates to ensue and likely to be unsolved as embryoid research advances.

Our answers to these and other fundamental questions likely will lead to different judgments about what research may and may not be done using embryoids. If we were to assume that embryoids are or ought to be treated as if they were equivalent to human embryos, ongoing disagreements about the permissibility of embryo research would apply here as well, with some advocating for different limits or restrictions and others advocating to expand such research.

### Public trust, engagement, transparency and research hype

Many publications mention the importance of trust, public or stakeholder engagement, and/or transparency. However, they rarely define engagement or transparency or indicate how it could be achieved. Often, they include little or no discussion of who counts as a stakeholder and how they understand “the public” with whom they recommend engagement. There is no clarity about the type and scope of engagement they recommend nor the purpose of engagement. In addition, there is little to no discussion on how such engagement should inform research and policy decisions. For instance, Lovell-Badge et al. describe new guidelines banning genetic editing of embryos and note that “[i]t will also require meaningful public engagement, political support, and proper oversight within the relevant jurisdiction” [69]. However, neither the paper nor the ISSCR guidelines it references offers any details regarding who this public engagement should include, what it should address, the approach, methods, or models for such engagement, or how it should be used. It is also unclear how researchers would respond if public engagement results in recommendations for limiting or even restricting research.

Identifying stakeholders is an important first step toward a more robust account of public engagement regarding embryoid research. Stakeholders might include scientists, public and private funders of research, donors, patients and their families, patient advocacy organizations, policy makers, and regulators. At a broader level, members of the public in general are stakeholders since a large portion of biomedical research, especially in the USA, is publicly funded [28]. As a result, many believe that this research should be accountable to the public and that researchers should justify the use of limited resources for their work [28, 82]. For example, in the USA there is no stated priority list for research. However, the fact that the federal government invests significant resources into biomedical research, compared to other area of science, suggests that it is a major research priority. In contrast, human embryo research is not a public funding priority area in the USA and federal funding has been explicitly banned for several decades on all human embryo research [28]. The goals of public and stakeholder engagement often are not articulated, yet engagement goals should guide the methods and scope for such engagement [82].

Goals for stakeholder engagement regarding embryoid research remain unclear. ISSCR promotes public engagement for human embryo and embryoid research and has a Public Engagement Task Force [69, 83]. While ISSCR had limited stakeholder engagement when developing their guidelines, others, such as the American College of Obstetric and Gynecology, removed their 14-day limit recommendation without public comment [84]. However, engagement implies two-way communication—all parties both express their views and listen to others. If public input was not an important part of establishing policies, guidelines, or recommendations, it seems that they may be using the term “engagement” to refer to what might best be described as “public outreach.” Outreach involves scientists informing the public about the new policies and research, not listening to and considering various views [82]. True public engagement could help uncover broader social concerns related to research and ways to address them. These discussions, in turn, could play a key role in guiding future research or fostering public acceptance of new research.

As the science of embryoid research advances and these entities become more sophisticated, new questions likely will emerge. Recent work using mouse cells to create embryoids that grew in culture to the point limbs and organs was developing, including a beating primitive heart structure [10]. This work could challenge claims that embryoids will not be able to further develop in culture [10].

Overall, the process of assessing embryoid research should be iterative and therefore public and stakeholder engagement should be iterative. Scientists should consider engaging the public before the work becomes discussed extensively in the public forum, including the lay press, to foster an informed narrative. Ongoing engagement is important, particularly in light of recent significant changes to human embryo research guidelines that were adopted without robust public engagement [27, 57].

One possible explanation for the failure to engage the public is fear. Transparency, while often hailed as an important factor in building and maintaining public trust, could undermine scientists’ interest in garnering public support for embryoid research. Transparency about the significant uncertainty regarding potential future benefits of the research and whether it will yield knowledge that transforms and saves lives could dampen public support for the research. Perhaps to secure support for their work, scientists sometimes “overpromise” or hype as some scholars have suggested happened with hESC research [85]. Transparency, rather than inflated speculation, could weaken rather than strengthen public support. Similarly, transparency about the potential to develop sophisticated entities that could be models of later stage embryos as well as fetuses could interfere with public support or result in unwanted regulatory oversight.

Thus far, scientists have emphasized that embryoids do not have the potential to develop and model later stage human embryos and fetuses [47]. These remarks might be meant, in part, to assuage fears or concerns about this research that could undermine support for it. However, we do not know the true potential of many models until their limits are tested. Significant developments in the area of human embryo research as well as developments in mouse embryoid research suggest that these entities will continue to become more sophisticated [10]. There is no mechanism of accountability in place to avoid the creation of embryoids that mimic later developmental stages or with advanced neurological systems. ISSCR’s recent decision to remove the 14-day limit on human embryo and embryoid research in its guidelines results in having no developmental or time limit for such work. Given the recent incident developing humans with permanent germline edits prior to public or scientific acceptance of this work, guidelines without enforcement are likely to do little to limit researchers interested in expanding to new areas [86]. It is therefore plausible that scientists would see no reason to set a firm limit on the developmental stages or features of embryoids.

Despite numerous calls for public and stakeholder engagement and transparency regarding embryoid research in the publications we reviewed, very little substantive work has been done in this area.

### Guidance, oversight, or regulation

Many (*n* = 21, 37%) publications called for “something” to guide embryoid research. Some authors suggest that ethical guidance will suffice, while others indicate that oversight is or will become necessary as science advances and embryoids resemble embryos more closely. Although some scholars offered recommendations for how embryoid research should be conducted, the scope of any possible guidance remains largely undefined. Existing stem cell research guidelines either do not mention embryoids [87] or provide significant flexibility regarding recommended oversight [69]. One’s understanding of what embryoids are and the values and goals one prioritizes likely will shape the kind of guidance, oversight, or regulation one deems appropriate.

To determine whether and how embryoid research should be regulated or overseen and what such oversight or regulation should require, permit, or prohibit requires, clarity regarding the purpose of regulation and oversight as well as clear definition of what is and is not acceptable will be necessary. Oversight could aim at building and maintaining trust, avoiding wrongdoing, facilitating research, or avoiding liability and ensuring compliance with funders’ and publishers’ policies. Many unanswered questions remain about who should develop regulations or policies, what they should permit and prohibit, how they should be enforced, and who should oversee such research and how.

Understandings of what kinds of entities embryoids are and their moral significance could shape assessments of appropriate regulatory and oversight mechanisms. Someone who sees them as no different from any other collection of cells or biological tissue will answer these questions differently from someone who views sophisticated embryoids as human embryos. Among the latter, views about oversight and limits will turn on their understanding of permissible research on human embryos.

### Lack of consensus

Research in this field has been rapidly expanding and the number of publications increased over time. Different areas of concerns were addressed, and recommendations were made by various authors on how to respond to concerns. We did not see any consensus-building efforts, nor did we see any consensus emerging within the field. Authors associated with the 2021 ISSCR guidelines promoted specific recommendations by ISSCR, including how to name and regulate embryoids [69]. However, several of these recommendations were challenged in subsequent literature, including what to call embryoids and how to define them [9, 88–90]. For example, a 2023 ISSCR statement expressed concern over the use of terms that suggest that embryo models are embryos [91].This lack of consensus suggests that scientists working in this area themselves do not agree on standards or how to move forward in uncertain regulatory climates.

### Limitations

There are several ways in which this systematic review did not capture every relevant publication on this topic. First, we searched only for publications in English, missing conversations on the subject, especially by Chinese researchers who are conducting embryo and embryoid research. Second, embryoid research is developing rapidly and this review reflects a snapshot of the literature up to the date on which we closed our search. Newer material that addresses more recent developments is not represented here. Finally, although the search was carefully designed by a multidisciplinary team that included research librarians, it is possible that we failed to locate some relevant publications. This is due in part to the wide range of terms that have been used to describe embryoids. Due to the nature of this review, we could not assess the quality or significance of the publications reviewed.

## Conclusion

Much work remains to be done to address the ethical, legal, regulatory, and policy considerations relevant to embryoid research. As this review revealed, thus far discussion of the risks or potential harms associated with this research has been quite limited and very little attention has been given to this topic from various religious perspectives. As this work becomes more widely known, it will be important to engage people of various faiths to understand how various religious frameworks view embryoids and to expand our understanding of the full range of relevant considerations. At this time, a critical question is how to operate with respect to embryoid research in the face of uncertainty and ongoing scientific developments.

## Data Availability

Datasets generated and analyzed during the current study are available from the first author upon request.

## Appendix 1: search strategy

### PubMed

(“Artificial embryo” OR embryoid OR “embryonic organoid” OR “SHEEF” OR “synthetic human entity with embryo-life features” OR “SHEEFs” OR “Synthetic embryo” OR “Blastoid” OR “Gastruloid” OR “PASE” OR “post-implantation amniotic sac embryoid” OR “Micropatterned hESC colony” OR “Micropatterned hESC colonies” OR “ETS Embryo” OR “ETX Embryo” OR “human cell-culture models of early development” OR “hCCMED”) AND (Ethics OR ethic OR ethical OR moral OR Policy OR policies OR Guideline OR Guidelines OR.

Recommendation OR Recommendations OR Law OR Legal OR Regulation OR Regulatory OR.

Oversight OR Governance).

Limits: English.

### Embase

(“Artificial embryo” OR embryoid OR “embryonic organoid” OR “SHEEF” OR “synthetic human entity with embryo-life features” OR “SHEEFs” OR “Synthetic embryo” OR “Blastoid” OR “Gastruloid” OR “PASE” OR “post-implantation amniotic sac embryoid” OR “Micropatterned hESC colony” OR “Micropatterned hESC colonies” OR “ETS Embryo” OR “ETX Embryo” OR “human cell-culture models of early development” OR “hCCMED”) AND (Ethics OR ethic OR ethical OR moral OR Policy OR policies OR Guideline OR Guidelines OR.

Recommendation OR Recommendations OR Law OR Legal OR Regulation OR Regulatory OR.

Oversight OR Governance).

Limits: English.

### Web of Science

(“Artificial embryo” OR embryoid OR “embryonic organoid” OR “SHEEF” OR “synthetic human entity with embryo-life features” OR “SHEEFs” OR “Synthetic embryo” OR “Blastoid” OR “Gastruloid” OR “PASE” OR “post-implantation amniotic sac embryoid” OR “Micropatterned hESC colony” OR “Micropatterned hESC colonies” OR “ETS Embryo” OR “ETX Embryo” OR “human cell-culture models of early development” OR “hCCMED”) AND (Ethics OR ethic OR ethical OR moral OR Policy OR policies OR Guideline OR Guidelines OR.

Recommendation OR Recommendations OR Law OR Legal OR Regulation OR Regulatory OR.

Oversight OR Governance).

Limits: English.

## Abbreviations

dpf: Days post-fertilization
hER: Human embryo research
hESC: Human embryonic stem cell research
ISSCR: International Society for Stem Cell Research
